# The Longitudinal Association Between Parent-Child Attachment and Adolescent Depressive Symptoms: Moderation by Oxytocin Receptor Polymorphisms

**DOI:** 10.3390/bs16071259

**Published:** 2026-07-22

**Authors:** Xiujin Lin, Wanyu Ye, Yuzhe He, Pei Chen, Jian Mao, Yingying Lai, Wenhao Gu, Yiling Luo, Shengnan Li, Yangang Nie

**Affiliations:** Research Center of Adolescent Psychology and Behavior, Department of Psychology, School of Education, Guangzhou University, Guangzhou 510006, China; lin.xiujin00@gzhu.edu.cn (X.L.); 2112108042@e.gzhu.edu.cn (W.Y.); 2112208038@e.gzhu.edu.cn (Y.H.); 1112108001@e.gzhu.edu.cn (P.C.); 1112308001@e.gzhu.edu.cn (J.M.); 2112408158@e.gzhu.edu.cn (Y.L.); gwhpsy@e.gzhu.edu.cn (W.G.); 2112408120@e.gzhu.edu.cn (Y.L.)

**Keywords:** parent-child attachment, OXTR, depressive symptoms, adolescents, moderation, longitudinal study

## Abstract

Maternal and paternal attachment may relate differently to adolescent depressive symptoms, but the potential contribution of oxytocin receptor (OXTR) gene single nucleotide polymorphisms (SNPs) to these associations remains unclear. This six-month longitudinal study included 746 seventh- and tenth-grade students (50.7% female) from Guangzhou, China. Parent-child attachment and saliva samples for genotyping were collected at baseline, and depressive symptoms were assessed at follow-up using the CES-D-10. Overall, 48.8% of adolescents screened positive for depressive symptoms. Compared with adolescents below the cutoff, those who screened positive reported lower parental trust/communication and higher alienation (all *p* < 0.01). In adjusted linear regression models, father-child trust/communication was negatively associated with subsequent depressive symptoms (*β* = −0.26, *p* < 0.01), whereas mother-child alienation was positively associated with subsequent depressive symptoms (*β* = 0.15, *p* = 0.02). Exploratory moderation analyses showed a nominal rs2254295 × mother-child alienation interaction before correction (*β* = −0.07, *p* = 0.046), but this effect did not survive FDR correction. Father-child trust/communication and mother-child alienation showed distinct associations with adolescent depressive symptoms. The possible moderating role of OXTR rs2254295 should be regarded as preliminary and requires replication in larger independent samples.

## 1. Introduction

### 1.1. Depressive Symptoms

Depressive symptoms, characterized by persistent low mood and related cognitive, emotional, and behavioral difficulties, are a major mental health concern in adolescence (Beck, 1967; Gotlib & Hammen, 2008). A recent national report indicated that approximately 14.8% of Chinese adolescents show varying levels of depression risk (Fu & Zhang, 2023). Depressive symptoms during adolescence are associated with suicide risk and impairments in cognitive functioning, emotion regulation, social engagement, and academic performance (Jia et al., 2018). They may also persist into adulthood and contribute to later mental and physical health problems and psychosocial impairment (Psychogiou et al., 2024; W. Zhang & Wang, 2015), generating substantial social and economic burden (Farrell et al., 2024; Racine et al., 2021; Whiteford et al., 2013). Identifying relational and biological factors associated with adolescent depressive symptoms may therefore inform more targeted prevention and intervention efforts.

### 1.2. Parent-Child Attachment and Depressive Symptoms

Gene-environment interaction (G × E) models offer a useful framework for examining how genetic liability and environmental experience may jointly contribute to adolescent depressive symptoms (D. W. Belsky & Domingue, 2023). Bronfenbrenner’s bioecological model likewise emphasizes the importance of repeated interactions within proximal developmental contexts (Bronfenbrenner & Morris, 1998). During adolescence, parent-child attachment is one such context, as everyday interactions with caregivers may shape emotional security, interpersonal expectations, and vulnerability to depressive symptoms. Attachment theory proposes that caregiver experiences are internalized as working models of the self and others, including beliefs about whether the self is worthy of care and whether others are available and responsive (Bowlby, 1969; Fearon et al., 2016; M. Wang et al., 2024). Secure attachment can provide a secure base and safe haven that support coping and emotion regulation, whereas insecure attachment may involve anxiety, avoidance, alienation, or expectations of caregiver unavailability or rejection (Bowlby, 1969; Mikulincer & Shaver, 2016). Recent work further suggests that attachment-related risk may be linked to depressive symptoms through emotion awareness and emotion regulation processes (Carapeto et al., 2022; Iwanski et al., 2021; Lin et al., 2024).

Although attachment is often described globally as secure or insecure (Li & Zhang, 2013; Rogers et al., 2022; Zimmermann et al., 2009), adolescent parent-child attachment can also be examined through specific relational dimensions. The Inventory of Parent and Peer Attachment assesses perceived attachment quality through trust, communication, and alienation (Armsden & Greenberg, 1987). Trust and communication reflect supportive features of the parent-adolescent relationship that may facilitate coping and emotion regulation, whereas alienation captures anger, emotional distance, and perceived disconnection. These dimensions may therefore relate to depressive symptoms in different ways. Prior studies have linked insecure attachment to depressive symptoms in children and adolescents (Spruit et al., 2020; Trucharte et al., 2022; Verhees et al., 2021), and recent longitudinal and mediation studies suggest that maternal and paternal attachment may contribute to depressive symptoms partly through emotion regulation (Carapeto et al., 2022; Iwanski et al., 2021; Lin et al., 2024). These findings support the view that attachment is a developmentally meaningful risk or protective factor rather than a deterministic cause. Because adolescents may form distinct attachment relationships with mothers and fathers (Bowlby, 1969), it remains important to examine whether specific maternal and paternal attachment dimensions show different longitudinal associations with depressive symptoms.

### 1.3. OXTR Polymorphisms, Parent-Child Attachment, and Depressive Symptoms

The differential susceptibility model is one of the main theoretical perspectives in G × E research (J. Belsky, 1997). Oxytocin has been implicated in social functioning and mood-related processes (Cochran et al., 2013). Oxytocin is a nonapeptide released by the hypothalamic paraventricular and supraoptic nuclei and is involved in labor, lactation, prosocial behavior, and adaptive social functioning (Sharma et al., 2020). The oxytocin receptor gene (OXTR), a central component of the oxytocin signaling pathway, has therefore been studied as a biologically plausible candidate gene in relation to social bonding, attachment-related behavior, social cognition, and stress regulation. However, evidence linking OXTR polymorphisms to socio-emotional and behavioral functioning in children and adolescents remains heterogeneous (Kohlhoff et al., 2022). Previous studies have also examined OXTR polymorphisms in relation to mental disorders, including depression (Pierzynowska et al., 2023; Smearman et al., 2016), and the oxytocin system has been discussed as a possible biological pathway underlying attachment-related processes (Bakermans-Kranenburg & van IJzendoorn, 2014; Bosmans et al., 2020; F. S. Chen et al., 2012). A review further suggested that rs53576 and rs2254298 may be involved in G × E processes related to depression and anxiety symptoms (Cataldo et al., 2018). Nevertheless, the role of oxytocin signaling in the association between parent-child attachment and adolescent depressive symptoms remains unclear. Given the small and inconsistent effects reported in candidate-gene studies, the present OXTR analyses were treated as exploratory tests of biological sensitivity rather than confirmatory evidence of genetic susceptibility (D. W. Belsky & Domingue, 2023; Border et al., 2019; Plomin et al., 2022).

The present study had two aims. First, we examined whether maternal and paternal attachment dimensions were differentially associated with adolescent depressive symptoms over a six-month period. We hypothesized that higher parental trust/communication would be associated with lower depressive symptoms, whereas higher parental alienation would be associated with higher depressive symptoms. Second, we explored whether selected OXTR polymorphisms (rs53576, rs2254295, rs2254298, and rs2268493) moderated the associations between parent-child attachment dimensions and depressive symptoms. Because prior evidence regarding single OXTR polymorphisms is mixed and candidate-gene effects are typically small, these moderation analyses were considered exploratory and hypothesis-generating.

## 2. Materials and Methods

### 2.1. Participants and Procedures

**Figure 1:**
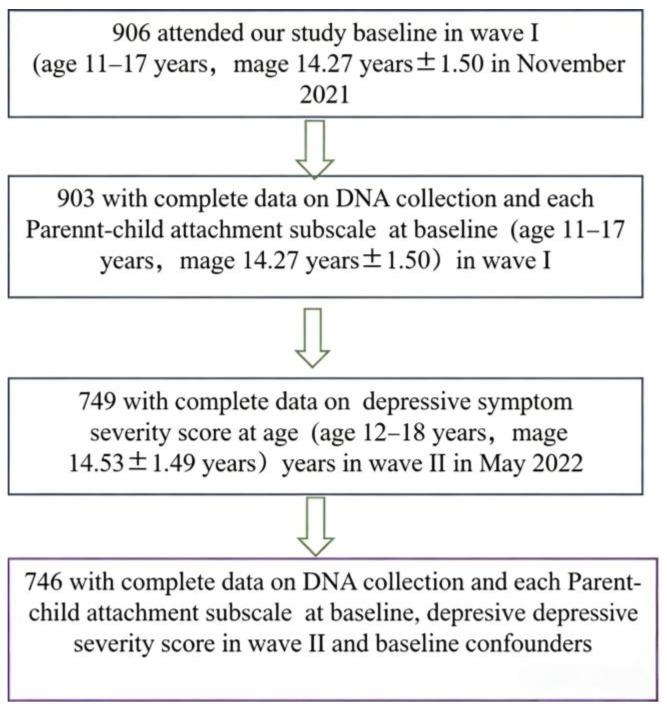
Research flowchart.

This prospective cohort study used data collected at two time points. At Wave I (November 2021), participants were aged 11–17 years (M = 14.27 years), and parent-child attachment and saliva samples for DNA extraction were collected. At Wave II (May 2022), participants were aged 12–18 years (M = 14.53 years), and depressive symptoms were assessed. At baseline, 903 participants had data on DNA collection and each parent-child attachment subscale. The final analytic sample included 746 adolescents with valid data on baseline DNA collection, baseline parent-child attachment, Wave II depressive symptoms, and Wave II demographic covariates (Figure 1). Parent-child attachment was assessed by adolescent self-report using the Inventory of Parent and Peer Attachment (IPPA-R); therefore, the analytic sample for both maternal and paternal attachment comprised the same 746 adolescents.

Ethical approval was obtained from the Institutional Review Board of Guangzhou University (Approval No. GZHU2019007). Written informed consent was obtained from participants’ guardians, and verbal assent was obtained from all adolescents. Participants were informed that they could withdraw at any time without penalty. Trained research assistants administered the assessments in classrooms during regular school hours according to a standardized protocol. No compensation or incentive was provided.

### 2.2. Survey Tools

#### 2.2.1. Demographic Information

Demographic information was collected using a self-administered questionnaire, including sex (1 = male, 2 = female), age, maternal and paternal education (low = primary school, secondary school, high school, or no formal education; high = college degree or above), weekly cohabitation time with parents (≥5 days vs. <5 days), and parental employment status (one or both parents working outside vs. neither parent working outside).

#### 2.2.2. Inventory of Parent and Peer Attachment (IPPA-R)

The Chinese adaptation of the IPPA-R was developed by S. Wang and Su (2012) based on the original IPPA by Armsden and Greenberg (1987). The adapted scale includes paternal attachment, maternal attachment, and peer attachment subscales, with 10 items in each subscale assessing communication, trust, and alienation. In the present study, trust and communication were combined into a single trust/communication dimension, consistent with the scoring structure used in the main analyses. Confirmatory factor analyses supported the two-factor structure of trust/communication and alienation for both father-child attachment, χ^2^(34) = 206.18, CFI = 0.949, TLI = 0.932, RMSEA = 0.082, SRMR = 0.046, and mother-child attachment, χ^2^(34) = 169.86, CFI = 0.958, TLI = 0.945, RMSEA = 0.073, SRMR = 0.044. Internal consistency was acceptable in the current sample, with Cronbach’s α ranging from 0.797 to 0.876 across the four subscales.

#### 2.2.3. Depressive Symptoms (10-Item Center for Epidemiologic Studies Depression Scale, CES-D-10)

Depressive symptoms were assessed using the 10-item Center for Epidemiologic Studies Depression Scale (CES-D-10). The original CES-D was developed by Radloff (1977), and the 10-item short form was later evaluated by Andresen et al. (1994). The CES-D-10 is a widely used self-report screening measure of depressive symptoms. Items are rated on a four-point Likert scale from 0 to 3, yielding total scores from 0 to 30; higher scores indicate more severe depressive symptoms. A score of 10 or above is commonly used to indicate positive screening for depressive symptoms, and this cutoff has been validated and widely applied in Chinese adolescent samples (Andresen et al., 1994; Yang et al., 2018). The CES-D-10 has shown good reliability and structural validity among Chinese adolescents (Yang et al., 2018).

### 2.3. Genotype Analysis

SNPs were selected on the basis of prior literature and two criteria: (i) involvement in biological pathways relevant to psychological distress in clinical or nonclinical populations and (ii) documented OXTR variation in Han Chinese populations (HapMap database: http://www.hapmap.org, accessed on 21 July 2022). Because this study was hypothesis-driven, we used a candidate-gene approach focusing on OXTR polymorphisms with theoretical relevance to social bonding, attachment, and stress-related emotional functioning. The target variants were rs53576, rs2254295, rs2254298, and rs2268493. Genotyping followed routine polymerase chain reaction (PCR) protocols described in our previously published work (Liang et al., 2024). Alleles were amplified and discriminated using the improved multiplex ligase detection reaction (iMLDR). Dominant, recessive, and additive models were examined for each SNP. The genetic coding was as follows: rs53576, dominant model (GG/GA vs. AA), recessive model (GG vs. GA/AA), and additive model (per G allele); rs2254295, dominant model (TT/TC vs. CC), recessive model (CC vs. TC/TT), and additive model (per C allele); rs2254298, dominant model (GG/GA vs. AA), recessive model (GG vs. GA/AA), and additive model (per G allele); and rs2268493, dominant model (CC/CT vs. TT), recessive model (CC vs. CT/TT), and additive model (per C allele). Because none of the four OXTR SNPs showed direct associations with depressive symptoms, a polygenic risk score was not constructed; instead, gene-environment interactions were examined.

### 2.4. Statistical Analysis

All analyses were conducted in SPSS Statistics version 23.0 with a two-tailed significance level of *p* < 0.05. Descriptive statistics were calculated for demographic and study variables. Hardy-Weinberg equilibrium (HWE) for each OXTR SNP was tested using chi-square tests. Attrition analyses compared adolescents included in the final analytic sample with those lost to follow-up on demographic and baseline attachment variables. Multiple linear regression was used to examine the main effects of parent-child attachment and OXTR genotypes on Wave II depressive symptoms. All models were adjusted for Wave II sex, age, maternal and paternal education, weekly cohabitation time with parents, and parental employment status. Because depressive symptoms were not assessed at baseline, baseline depressive symptoms could not be included as a covariate; accordingly, the analyses estimated associations with subsequent depressive symptoms rather than changes in depressive symptoms over time. Parent-child attachment variables included total attachment scores and specific dimensions for maternal and paternal attachment. OXTR genotypes were entered as categorical predictors. Moderation analyses were conducted using PROCESS Model 1 with 5000 bootstrap samples. Separate models were fitted for each OXTR SNP and parent-child attachment variable, with dominant, recessive, and additive genetic models examined for each SNP. To address multiple interaction testing, false discovery rate (FDR) correction using the Benjamini-Hochberg procedure was applied to the SNP × attachment terms examined in the moderation analyses. Genetic moderation findings were interpreted as exploratory rather than confirmatory.

## 3. Results

### 3.1. Demographic Information Analysis

The final analytic sample included 746 adolescents (response rate = 83.0%). Of these participants, 379 (50.7%) were female, 396 (53.0%) lived with their parents for more than five days per week, and 662 (88.6%) had one or both parents working outside the home. In addition, 351 fathers (47.0%) and 355 mothers (47.5%) had a high education level, defined as a college degree or above. Genotype frequencies and sample characteristics are shown in Table 1.

Attrition analyses indicated that, among the 902 adolescents with complete baseline parent-child attachment data, 156 were lost to follow-up, yielding an attrition rate of 17.3%. Participants retained in the final analytic sample differed from those lost to follow-up on several demographic and baseline attachment-related variables, suggesting that attrition was not completely random (see Supplementary Table S1).

Genotype frequencies for OXTR rs53576, rs2254295, rs2254298, and rs2268493 did not deviate from HWE in the total sample (*p* > 0.05).

### 3.2. Parent-Child Attachment, OXTR Polymorphisms, and Adolescent Depressive Symptoms

A CES-D-10 score of 10 or higher was used to indicate positive screening for depressive symptoms. In the final sample, 364 adolescents (48.8%) screened positive. As shown in Table 2, adolescents who screened positive reported lower father-child and mother-child trust/communication than those below the cutoff (all *p* < 0.01). They also reported higher father-child and mother-child alienation (all *p* < 0.01).

Multiple linear regression analyses showed that the total parent-child attachment score was negatively associated with subsequent depressive symptoms (*β* = −0.34, 95% *CI* [−0.33, −0.16], *p* < 0.01; Table 3). Father-child trust/communication was also negatively associated with subsequent depressive symptoms (*β* = −0.26, 95% *CI* [−0.37, −0.16], *p* < 0.01), whereas mother-child alienation was positively associated with subsequent depressive symptoms (*β* = 0.15, 95% *CI* [0.04, 0.40], *p* = 0.02). No significant main effects were observed for OXTR rs53576, rs2254295, rs2254298, or rs2268493 (Table 3).

### 3.3. Exploratory Moderation Analyses of OXTR Polymorphisms

We examined whether OXTR rs53576, rs2254295, rs2254298, and rs2268493 moderated the association between parent-child attachment and adolescent depressive symptoms after adjustment for Wave II demographic covariates. In the uncorrected analyses, OXTR rs2254295 under the dominant model (CT + TT vs. CC) showed a nominal moderating effect on the association between mother-child alienation and depressive symptoms (*β* = −0.07, 95% *CI* [−0.60, −0.01], *p* = 0.046; Table 4). This model was adjusted for Wave II sex, age, maternal and paternal education, weekly cohabitation time with parents, parental employment status, and other types of parent-child attachment. However, this effect did not survive FDR correction and should therefore be considered exploratory rather than confirmatory.

**Figure 2:**
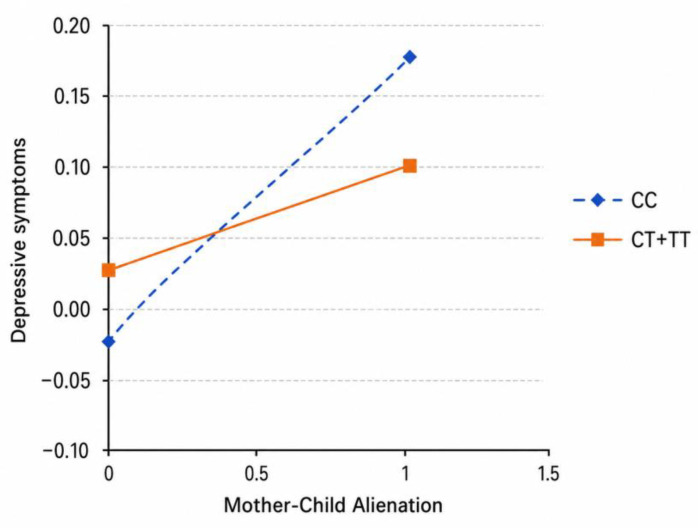
Exploratory plot of the OXTR rs2254295 × mother-child alienation interaction in predicting depressive symptoms.

For descriptive purposes, the interaction pattern suggested that the positive association between mother-child alienation and depressive symptoms was more pronounced among adolescents with the OXTR rs2254295 CC genotype and attenuated among T-allele carriers (*β* = 0.49; 95% *CI* = [0.16, 0.81], *p* = 0.003; Figure 2).

No other OXTR SNP × attachment interaction remained significant after FDR correction. Estimates for all tested genetic models are provided in Supplementary Tables S2–S5.

## 4. Discussion

This prospective longitudinal study examined the distinct associations of maternal and paternal attachment with adolescent depressive symptoms and explored whether these associations were moderated by OXTR polymorphisms. The results suggest that father-child trust/communication and mother-child alienation were differentially associated with subsequent depressive symptoms. The rs2254295-related moderation effect emerged only as a nominal association in uncorrected exploratory analyses and did not survive FDR correction; therefore, it should not be interpreted as evidence of a robust genetic moderation effect.

The positive screening rate for depressive symptoms was high in this sample: 364 of 746 adolescents scored 10 or above on the CES-D-10, corresponding to a rate of 48.8%. This figure should be interpreted cautiously because the CES-D-10 is a self-report screening measure rather than a diagnostic instrument. The timing of data collection may also be relevant. Data were collected during the COVID-19 pandemic, when depressive symptoms among children and adolescents increased worldwide (Ma et al., 2021; Racine et al., 2021). Evidence from Chinese children and adolescents similarly indicates a substantial burden of depressive symptoms during this period (J. Chen et al., 2022). Disruptions to school routines, reduced peer contact, social isolation, uncertainty, and increased academic or family stress may have contributed to the elevated screening rate (Loades et al., 2020). Thus, the present rate should be understood as an indicator of depressive symptom burden in a specific pandemic context, rather than as the prevalence of clinically diagnosed depression.

Consistent with prior work, adolescents who screened positive for depressive symptoms reported higher mother-child alienation and lower trust/communication with both parents (Ebbert et al., 2019; Q. Zhang et al., 2021). This pattern also aligns with evidence that family communication interventions can reduce anxiety and depressive symptoms (Lloyd et al., 2023). A more specific finding was that father-child trust/communication was negatively associated with subsequent depressive symptoms. This is consistent with studies linking higher-quality father-child relationships to better adolescent emotional adjustment (Xu et al., 2023). Fathers may provide relational experiences that complement maternal caregiving, including support for autonomy, exploration, and coping with challenges. Conversely, mother-child alienation was positively associated with subsequent depressive symptoms, in line with work linking lower-quality maternal relationships and alienation-related experiences with poorer emotional adjustment (Harman et al., 2022; Verrocchio et al., 2018), including low self-esteem and depressive or anxiety symptoms (Sun et al., 2021; Verrocchio et al., 2016). Mother-child alienation may reflect perceived emotional distance, reduced support, and lower relational security within the mother-child relationship. Together, these findings are consistent with the multiple-attachment perspective, which holds that children can form distinct and complementary relationships with different caregivers, each with unique developmental implications (Ainsworth & Bowlby, 1991).

The genetic moderation findings should be interpreted cautiously. In uncorrected exploratory analyses, the rs2254295 × mother-child alienation term showed a nominal association with depressive symptoms; however, this effect did not survive FDR correction. Therefore, this finding should be regarded as a preliminary statistical signal rather than evidence of a reliable genetic moderation effect. Although the interaction pattern was descriptively consistent with a possible genotype-dependent difference in the association between mother-child alienation and depressive symptoms, this interpretation remains uncertain and requires replication in larger independent samples.

Previous studies have suggested that the T allele of OXTR rs2254295 may be associated with increased OXTR expression (Bozorgmehr et al., 2019), and oxytocin-related pathways have been implicated in stress reactivity and socio-emotional functioning through their connections with hypothalamic-pituitary-adrenal (HPA) axis activity (Jiang et al., 2023). However, the present study did not directly assess oxytocin levels, OXTR expression, receptor density, neural activity, or HPA-axis functioning. Therefore, the proposed biological explanation should be regarded as a theoretical possibility rather than as direct empirical evidence.

The other three OXTR SNPs examined (rs53576, rs2254298, and rs2268493) showed no significant main effects on depressive symptoms and no significant moderation effects in the attachment-depression association. These null findings are broadly consistent with prior evidence. Meta-analytic work suggests that rs53576 and rs2254298 explain little variation in human social behavior (Bakermans-Kranenburg & van IJzendoorn, 2014), and a three-approach study including meta-analyses found no association between these SNPs and attachment dimensions (Gong et al., 2020). One possible explanation is functional specificity, whereby different OXTR SNPs may be linked to different socio-emotional pathways (León-Rodríguez et al., 2024). Another possibility is limited power: detecting small G × E effects typically requires larger samples than the present study. Future studies should use larger samples and preregistered genetic models.

### Strengths and Limitations

This study has several strengths, including its prospective longitudinal design, the separate examination of maternal and paternal attachment, and the inclusion of OXTR polymorphism analyses. The findings point to the importance of distinguishing father-child trust/communication from mother-child alienation when examining adolescent depressive symptoms. At the same time, the genetic moderation results are preliminary and should be interpreted with caution.

Several limitations should be noted. First, although the study used a prospective design, data were collected at only two time points, and depressive symptoms were assessed only at follow-up. Baseline depressive symptoms could not be controlled; therefore, the findings should be interpreted as associations between baseline attachment and subsequent depressive symptoms rather than evidence of change in depressive symptoms over time. Future studies should include repeated assessments of depressive symptoms across multiple waves. Second, the genetic analyses used a candidate-gene approach focused on four OXTR polymorphisms. Although theoretically grounded, candidate-gene findings are often limited by small effect sizes, mixed evidence, and replication difficulties. The nominal rs2254295 interaction also did not survive FDR correction; thus, the genetic moderation findings should be considered exploratory and require independent replication in larger samples using genome-wide, polygenic, or epigenomic approaches. Third, approximately 17% of adolescents with complete baseline parent-child attachment data were lost to follow-up. Attrition analyses suggested that attrition was not completely random, as retained participants differed from those lost to follow-up on several demographic and baseline attachment-related characteristics. Although key Wave II demographic covariates were adjusted for in the main analyses, potential attrition bias cannot be ruled out. Finally, attachment was assessed by adolescent self-report, which may be affected by recall bias and shared method variance. Multi-informant or interview-based assessment of attachment would help improve phenotypic precision in future research.

## 5. Conclusions

This study suggests that father-child trust/communication is associated with lower subsequent depressive symptoms, whereas mother-child alienation is associated with higher subsequent depressive symptoms. A nominal interaction between OXTR rs2254295 and mother-child alienation emerged in exploratory analyses, but it did not survive FDR correction and therefore requires cautious interpretation and replication. These findings underscore the value of distinguishing maternal and paternal attachment dimensions when studying adolescent depressive symptoms. Prevention and intervention efforts may benefit from strengthening parent-child relationships, whereas genetic moderation findings should be viewed as preliminary until replicated in larger independent samples.

## Figures and Tables

**Table 1:** Genotype frequencies of OXTR polymorphisms in the final analytic sample (*N* = 746).

SNP	Genotype	Derived/Ancestral Allele	*n*	*f*	Total	*p*-HWE
rs53576	AA/AG/GG	A/G; A>G,*T*	335/320/77	45.8/43.7/10.5	731	0.98
rs2254295	CC/CT/TT	*T*/C; *T*>A,C	63/335/337	8.6/45.6/45.8	733	0.13
rs2254298	AA/AG/GG	G/A; G>A,*T*	69/319/352	9.3/43.1/47.6	739	0.87
rs2268493	TT/CT/CC	C/*T*; *T*>C	533/179/11	73.7/24.8/1.5	722	0.36

Note. N = 746 refers to the final analytic sample. Valid genotyping sample sizes differed across SNPs because of genotype call failure or missing genotype data. The number of successfully genotyped participants was 731 for rs53576, 733 for rs2254295, 739 for rs2254298, and 722 for rs2268493. HWE = Hardy-Weinberg equilibrium.

**Table 2:** Group differences between adolescents with and without depressive symptoms (N = 746).

Variables	No Depressive Symptoms 382 (<10)	Depressive Symptoms 364 (≥10)	*t* (*p*)
FCA trust and communication	23.88 (5.48)	20.35 (5.92)	8.46 (<0.01)
MCA trust and communication	24.75 (4.74)	22.22 (6.09)	6.35 (<0.01)
FCA alienation	8.04 (4.07)	10.12 (4.00)	−7.06 (<0.01)
MCA alienation	7.77 (3.68)	9.86 (4.29)	−7.16 (<0.01)

Note. MCA = mother-child attachment; FCA = father-child attachment.

**Table 3:** Multivariable regression models predicting depressive symptoms at Wave II (N = 746).

	Independent Variables	Adjusted *β*	95% *CI*	*p*	*R*^2^ (*p*)
Model 1 (Parent-Child Attachment)	FCA-alienation	0.09	(−0.04, 0.31)	0.13	0.23 (<0.01)
	MCA alienation	0.15	(0.04, 0.40)	0.02	
	FCA trust and communication	−0.27	(−0.39, −0.18)	<0.01	
	MCA trust and communication	−0.003	(−0.11, 0.11)	0.96	
Model 2 (OXTR SNPs)	rs53576 (Additive model)	0.30	(−0.43, 1.03)	0.42	0.05 (<0.01)
	rs2254295 (Additive model)	0.05	(−1.47, 2.45)	0.62	
	rs2254298(Additive model)	0.08	(−1.19, 2.68)	0.45	
	rs2268493(Additive model)	−0.03	(−1.42, 0.65)	0.47	

Note. Models were adjusted for Wave II sex, age, maternal and paternal education, weekly cohabitation time with parents, and parental employment status. MCA = mother-child attachment; FCA = father-child attachment.

**Table 4:** Exploratory moderated regression model predicting depressive symptoms at Wave II (*N* = 746).

Independent Variables	Adjusted β	95% *CI*	*p*
MCA alienation	0.14	(0.03, 0.39)	0.02
rs2254295 (CT + TT/CC)	0.03	(−0.73, 2.16)	0.33
MCA alienation × rs2254295 (CT + TT/CC)	−0.07	(−0.60, −0.01)	0.046

Note. The model was adjusted for Wave II sex, age, maternal and paternal education, weekly cohabitation time with parents, parental employment status, and other types of parent-child attachment. MCA = mother-child attachment. The *p* value for the interaction term is uncorrected; the interaction did not remain significant after FDR correction and should be interpreted as exploratory.

## Data Availability

The data that support the findings of this study are available from the corresponding author upon reasonable request.

## Supplementary Materials

The following supporting information can be downloaded at: https://www.mdpi.com/article/10.3390/bs16071259/s1, Table S1: Differences between included participants and those lost to follow-up; Table S2: Moderated Regression Analyses Predicting Depression from PCAFT1 with rs53576 Genotype as Moderator; Table S3: Moderated Regression Analyses Predicting Depression from PCAFT1 with rs2254295 Genotype as Moderator; Table S4: Moderated Regression Analyses Predicting Depression from PCAFT1 with rs2254298 Genotype as Moderator; Table S5: Moderated Regression Analyses Predicting Depression from PCAFT1 with rs2268493 Genotype as Moderator.

## Abbreviations

The following abbreviations are used in this manuscript:

CES-D-10	10-item Center for Epidemiologic Studies Depression Scale
CI	Confidence interval
HWE	Hardy–Weinberg equilibrium
iMLDR	Improved multiplex ligase detection reaction
IPPA	Inventory of Parent and Peer Attachment
OXTR	Oxytocin receptor
PCR	Polymerase chain reaction
PRS	Polygenic risk score
SNP	Single nucleotide polymorphism
