# Supplementary material for: The Longitudinal Association Between Parent-Child Attachment and Adolescent Depressive Symptoms: Moderation by Oxytocin Receptor Polymorphisms
Source: Behav Sci (Basel). 2026 Jul 22;16(7):1259. doi: 10.3390/bs16071259 (PMC13405697; doi:10.3390/bs16071259)
Supplement: Supplementary file 1 [file behavsci-16-01259-s001.zip › behavsci-4394748-supplementary.pdf]

Table S1. Differences between included participants and those lost to follow-up

Variables	Included, n = 746	Lost to follow-up, n = 156	Test
Demographic characteristics			
Age, years	14.53 ± 1.49	15.41 ± 1.01	t = -8.79, p < .001
Female, n (%)	379 (50.7%)	45 (29.0%)	x^2 = 21.30, p < .001
Living with parents >5 days/week, n (%)	396 (53.0%)	17 (10.9%)	x^2 = 91.42, p < .001
One or both parents working outside, n (%)	662 (88.6%)	127 (81.4%)	x^2 = 4.05, p = .044
Father high education level, n (%)	351 (47.0%)	108 (69.2%)	x^2 = 18.48, p < .001
Mother high education level, n (%)	355 (47.5%)	88 (56.4%)	x^2 = 1.98, p = .160
**Baseline parent－child attachment**			
Father trust/communication	22.16 ± 5.97	23.12 ± 5.67	t = -1.90, p = .059
Mother trust/communication	23.51 ± 5.58	24.37 ± 4.90	t = -1.94, p = .053
Father alienation	9.06 ± 4.17	7.78 ± 3.12	t = 4.37, p < .001
Mother alienation	8.79 ± 4.12	7.71 ± 3.04	t = 3.77, p < .001

Table S2. Moderated Regression Analyses Predicting Depression from PCAFT1 with rs53576 Genotype as Moderator

Predictor	Model rs53576 (GG=1,GA=2,AA=3)		Model 2: rs53576 (GG=1,GA+AA=2)		Model 3: rs53576 (AA=1,GG+GA=2)	
	B	[95% CI]	B	[95% CI]	B	[95% CI]
FCA- trust and communication	-0.47 **	[-0.75, -0.19]	-0.45 ***	[-0.68, -0.22]	-0.47	[-0.95, 0.00]
rs53576	-1.01	[-3.62, 1.61]	-1.28	[-4.69, 2.14]	-1.21	[-6.93, 4.51]
PCAFT1 × rs53576	0.04	[-0.08, 0.15]	0.05	[-0.10, 0.20]	0.05	[-0.20, 0.30]

Note: adjusted for sex, age, Maternal and Paternal education, the time children spent with their parents, and Whether parents work outside.FCA: Father-Child attachment

Table S3. Moderated Regression Analyses Predicting Depression from PCAFT1 with rs2254295 Genotype as Moderator

Predictor	Model :rs2254295(CC=1，CT=2，TT=3)		Model 2: rs2254295(CC=1, CT+TT=2)		Model 3: rs2254295(TT=1, CT+CC=2)	
	B	[95% CI]	B	[95% CI]	B	[95% CI]
PCAFT1	-0.44	[-0.99,0.11]	-0.48***	[-0.72, -0.25]	-0.30*	[-0.59, -0.00]
rs2254295	0.02	[-6.70,6.73]	-1.66	[-5.04, 1.73]	1.07	[-1.67, 3.82]
PCAFT1 × rs2254295	0.03	[-0.25,0.32]	0.07	[-0.08, 0.21]	-0.04	[-0.15, 0.08]

Note: adjusted for sex, age, Maternal and Paternal education, the time children spent with their parents, and Whether parents work outside.FCA: Father-Child attachment

Table S4. Moderated Regression Analyses Predicting Depression from PCAFT1 with rs2254298 Genotype as Moderator

Predictor	Model: rs2254298 (GG=1,GA=2, AA=3)		Model 2: rs2254298 (AA=1, GG+GA=2)		Model 3: rs2254298 (GG=1,GA+AA=2)	
	B	[95% CI]	B	[95% CI]	B	[95% CI]
PCAFT1	-0.42**	[-0.71, -0.13]	-0.62*	[-1.14, -0.11]	-0.37**	[-0.60, -0.13]
rs2254298	-0.00	[-2.67, 2.66]	-2.13	[-8.46, 4.19]	0.60	[-2.77, 3.98]
PCAFT1 × rs2254298	0.01	[-0.10,0.13]	0.13	[-0.14, 0.39]	-0.01	[-0.16, 0.13]

Note: adjusted for sex, age, Maternal and Paternal education, the time children spent with their parents, and Whether parents work outside, FCA: Father-Child attachment

Table S5. Moderated Regression Analyses Predicting Depression from PCAFT1 with rs2268493 Genotype as Moderator

Predictor	Model :rs2268493(TT=1，CT=2，CC=3)		Model 2: rs2268493 (TT=1, CT+CC=2)		Model 3: rs2268493 (CC=1, CT+TT=2)	
	B	[95% CI]	B	[95% CI]	B	[95% CI]
PCAFT1	-0.36**	[-0.56, -0.15]	-0.37**	[-0.59, -0.14]	-0.22	[-0.78, 0.33]
rs2268493	0.34	[-2.93,3.62]	0.29	[-3.34, 3.92]	1.83	[-9.94, 13.59]
PCAFT1 × rs2268493	-0.02	[-0.17,0.12]	-0.01	[-0.17, 0.14]	-0.16	[-0.69, 0.38]

Note: adjusted for sex, age, Maternal and Paternal education, the time children spent with their parents, and Whether parents work outside, FCA: Father-Child attachment
